# Comparison of CRISPR-Cas9-mediated megabase-scale genome deletion methods in mouse embryonic stem cells

**DOI:** 10.1093/dnares/dsac045

**Published:** 2022-11-30

**Authors:** Masayuki Miyata, Junko Yoshida, Itsuki Takagishi, Kyoji Horie

**Affiliations:** Department of Physiology II, Nara Medical University, Kashihara, Nara 634-8521, Japan; Department of Physiology II, Nara Medical University, Kashihara, Nara 634-8521, Japan; Department of Physiology II, Nara Medical University, Kashihara, Nara 634-8521, Japan; Department of Physiology II, Nara Medical University, Kashihara, Nara 634-8521, Japan

**Keywords:** CRISPR, Cas9, deletion, homology-directed repair, non-homologous end joining

## Abstract

The genome contains large functional units ranging in size from hundreds of kilobases to megabases, such as gene clusters and topologically associating domains. To analyse these large functional units, the technique of deleting the entire functional unit is effective. However, deletion of such large regions is less efficient than conventional genome editing, especially in cultured cells, and a method that can ensure success is anticipated. Here, we compared methods to delete the 2.5-Mb Krüppel-associated box zinc finger protein (KRAB-ZFP) gene cluster in mouse embryonic stem cells using CRISPR-Cas9. Three methods were used: first, deletion by non-homologous end joining (NHEJ); second, homology-directed repair (HDR) using a single-stranded oligodeoxynucleotide (ssODN); and third, HDR employing targeting vectors with a selectable marker and 1-kb homology arms. NHEJ-mediated deletion was achieved in 9% of the transfected cells. Inversion was also detected at similar efficiency. The deletion frequency of NHEJ and HDR was found to be comparable when the ssODN was transfected. Deletion frequency was highest when targeting vectors were introduced, with deletions occurring in 31–63% of the drug-resistant clones. Biallelic deletion was observed when targeting vectors were used. This study will serve as a benchmark for the introduction of large deletions into the genome.

## 1. Introduction

Recent progress in genome science has revealed large functional units in the genome— such as gene clusters, promoter–enhancer loops, and topologically associating domains—which range from several hundred kilobases to megabases in length.^1^ Dysregulation of these functional units can lead to human diseases.^2^ To understand the genome from the perspective of such large functional units, technologies that can reliably modify large genomic regions are required. The recently developed CRISPR-Cas9 system^3^ is a powerful method for this purpose. Before the development of this system, the flox system was used to introduce large deletions into the genome.^4,5^ In the flox system, loxP sites are first inserted on both sides of the region to be deleted, and then deletions are induced by recombination between loxP sites using Cre recombinase. This method has the advantage that phenotypes can be directly compared before and after deletion induction in the same cell or animal if the efficiency of recombination by Cre is sufficiently high. However, the time and effort required to insert each loxP site into the genome is a drawback of this approach. Additionally, before the development of the CRISPR-Cas9 system, genome modification was possible only in specific cell lines, such as mouse embryonic stem cells (ESCs). On the other hand, CRISPR-Cas9 can introduce large deletions in various cells and animals without the use of Cre. If a comparison of the phenotype before and after deletion is needed, as in the flox system, it is possible to introduce loxP sites into the genome using CRISPR-Cas9 and subsequently induce deletion with Cre.

Megabase-scale genome modifications using CRISPR-Cas9 have been reported by either microinjection of Cas9 and guide RNAs (gRNAs) into zygotes^6–9^ or by transfection of cultured cells.^10–12^ However, genome editing in cultured cells is less efficient than zygote microinjection, and thus requires additional steps to identify edited clones such as the use of selection markers or screening of large numbers of clones. Biallelic megabase-scale deletion is even more challenging in cultured cells but is unquestionably required to conduct phenotype analysis in cultured cells.

The distal region of mouse chromosome 4 contains a 2.5-Mb region within which a gene cluster of Krüppel-associated box zinc finger protein (KRAB-ZFP) genes resides.^12^ KRAB-ZFP genes are known to be transcriptional repressors of retrotransposons.^13^ The retrotransposons and KRAB-ZFP genes have diversified in both nucleotide sequence and copy number as a result of their arms race. The diversified KRAB-ZFP genes also function as regulators of endogenous genes.^13^ We considered the deletion of this 2.5-Mb KRAB-ZFP gene cluster as an experimental model for megabase-scale genomic deletion in cultured cells and compared three methods employing the CRISPR-Cas9 system: (1) non-homologous end joining (NHEJ), (2) homology-directed repair (HDR) using a single-stranded oligodeoxynucleotide (ssODN) donor, and (3) HDR using double-stranded targeting vectors. The results will serve as a benchmark for megabase-scale genomic deletion methods in cultured cells.

## 2. Materials and methods

### 2.1 Cell line and cell culture

The REC24-3 mouse ESC line, a derivative of the V6.5 mouse ESC line,^14^ was used in this study. REC24-3 contains the ERT2-iCre-ERT2 cassette^15^ at the Rosa26 locus,^16^ which was introduced by the same procedure described previously.^17^ The presence of the ERT2-iCre-ERT2 cassette is irrelevant to the purpose of this study. ESCs were cultured in a serum-containing medium composed of KnockOut DMEM (Thermo Fisher Scientific; 10829018) supplemented with 20% fetal bovine serum, non-essential amino acids, 0.1 mM 2-mercaptoethanol, and 1,000 U/ml leukaemia inhibitory factor (Millipore; ESG1107). Mitomycin C (MMC)-treated mouse embryonic fibroblasts (MEFs) were used as feeder cells.

### 2.2 Construction of the Cas9/sgRNA expression vectors

Cas9 and single-guide RNAs (sgRNAs) were expressed using pX330.^18^ Complementary oligonucleotides for each sgRNA (Supplementary Table S2) were annealed and cloned into the BbsI site of pX330.

### 2.3 Construction of the targeting vectors

The targeting vectors were constructed using the primers listed in Supplementary Table S2 as follows. A 1-kb genomic fragment upstream of the cleavage site of sgRNA1 was amplified by polymerase chain reaction (PCR) from C57BL/6J genomic DNA using the primers Zfp600-5HR1-F1 and Zfp600-5HR1-R1. The fragment was digested with KpnI and HindIII and cloned into the KpnI-HindIII site of pPGKneo-F2F (gift from Dr. K. Yusa) adjacent to the neo-selection cassette, resulting in pPGKneoF2F-Zfp600-5HR. Next, a 1-kb genomic fragment downstream of the cleavage site of sgRNA2 was PCR-amplified from C57BL/6J genomic DNA using primers Zfp600-3HR1-F1 and Zfp600-3HR1-R1. The fragment was digested with NotI and SacII and cloned into the NotI-SacII site of the pPGKneoF2F-Zfp600-5HR, which is located opposite to the first cloning site of the neo-selection cassette, resulting in the neo-targeting vector pZfp600-DEL-TV1-Neo. The HindIII-NotI neomycin resistance gene (neo) cassette of pZfp600-DEL-TV1 was replaced with the HindIII-NotI hygromycin resistance gene (hyg) cassette of pPGKhyg-F2F (gift from Dr. K. Yusa), resulting in the hyg-targeting vector pZfp600-DEL-TV1-Hyg.

### 2.4 Transfection

The TransFast transfection reagent (Promega; E2431) was used in all transfections. ESCs (2.5 × 10^5^) were mixed with 2.5 µg of DNA and 15 µl of TransFast in serum-containing medium in a total volume of 500 µl and plated onto one well of a 24-well plate seeded with MMC-treated MEFs. After 1 h, 1 ml of medium was added to the well, and the medium was replaced with fresh medium 10 h after transfection. After this step, different culture protocols were utilized depending on the purpose of the experiment as described below.

### 2.5 Comparison of the deletion protocols and detection of genomic inversions

#### 2.5.1 Method 1

On Day 0, ESCs (2.5 × 10^5^) were transfected with 1.125 µg of pX330-gRNA1, 1.125 µg of pX330-gRNA2, and 0.25 µg of the puromycin resistance gene expression vector (pPGKpuro). ESCs were selected with 1 µg/ml of puromycin from Day 1 to Day 3 to enrich transfected cells. After completing puromycin selection on Day 3, ESCs were dissociated with trypsin/EDTA and plated sparsely on MEFs without puromycin for single-cell cloning; the remaining cells were subjected to genomic DNA purification as a bulk control. On Day 11, ESC colonies were picked and divided into two groups: one for DNA preparation for PCR analysis and the other for continuous culture to make frozen stocks. PCR template was prepared by resuspending ESC pellets in 20 µl of water, heating at 95°C for 10 min and cooling to room temperature. Then, the ESC lysate was digested with Proteinase K (5 µl of 2 mg/ml stock added) at 56°C for 60 min and then heat inactivated at 95°C for 10 min. After cooling to room temperature, 2 µl of ESC lysates were analysed by PCR using KOD FX polymerase (TOYOBO; KFX-101) with primers shown in Supplementary Table S2. Genomic deletion and inversion were detected by PCR with the following conditions: 94°C for 2 min for one cycle, followed by 35 cycles of 98°C denaturation for 10 s, 55°C annealing for 30 s. and 68°C extension for 30 s, and final extension at 68°C for 1 min. For the amplification of the Klf2 gene region as an internal control, the extension time of the PCR cycle and the final extension time after the PCR cycle were set to 1.5 and 3 min, respectively. When sequencing PCR products, PCR products were purified using QIAquick PCR Purification Kit (Qiagen; 28104) and sequenced directly without cloning to avoid the effect of errors caused by PCR. To investigate the reproducibility of deletion efficiency, three independent transfections were performed, and genomic DNA was purified from the bulk cell population on Day 3. PCR was conducted with the same conditions as described, except that the number of PCR cycles was decreased as shown in Fig. 2g to avoid saturating the PCR amplification.

#### 2.5.2 Method 2

On Day 0, ESCs (2.5 × 10^5^) were transfected with 0.75 µg of pX330-gRNA1, 0.75 µg of pX330-gRNA2, 0.75 µg of ssODN, and 0.25 µg of pPGKpuro. The remaining procedure is the same as in Method 1.

#### 2.5.3 Method 3

On Day 0, ESCs (2.5 × 10^5^) were transfected with 0.625 µg of pX330-gRNA1, pX330-gRNA2, pZfp600-DEL-TV1-Hyg, and pZfp600-DEL-TV1-Neo. On Day 1, ESCs were dissociated with trypsin/EDTA, plated onto 6-cm dishes, and subjected to three different drug selections: G418 only (150 μg/ml), hygromycin only (75 μg/ml), and G418 plus hygromycin (150 and 75 μg/ml, respectively). On Day 9, ESC colonies were picked and divided into two groups: one for lysate preparation for PCR analysis of the deletion events and the other for cell culture to make frozen stocks. PCR was conducted with the primers shown in Supplementary Table S2 with the same conditions as in Method 1 except that the extension time of the PCR cycle and the final extension time after the PCR cycle were set to 1.5 and 3 min, respectively. To investigate the reproducibility of deletion efficiency, three independent transfections were performed and genomic DNA was purified from the bulk cell population on day 9. The number of PCR cycles was decreased as shown in Fig. 4e to avoid saturating the PCR amplification.

### 2.6 Analysis of indels at the sgRNA target sites

ESC lysates, prepared in Methods 1 and 2 as described in the previous section, were analysed by PCR with the primers shown in Supplementary Table S2. PCR products were purified using QIAquick PCR Purification Kit and sequenced directly without cloning to avoid the effect of errors caused by PCR.

### 2.7 Single nucleotide polymorphism analysis

Genomic location of the single nucleotide polymorphism (SNPs) analysed for the allele heterozygosity at the Tnfrsf8 and the Miip loci are chr4:145268724 and chr4:147862304, respectively (GRCm38/mm10). Each genomic locus, including sequence flanking the target SNP, was amplified by PCR using KOD FX with primers shown in Supplementary Table S2. The PCR conditions were as follows: 94°C for 2 min for 1 cycle, followed by 35 cycles of 98°C denaturation for 10 s, 55°C annealing for 30 s, and 68°C extension for 30 s, and final extension at 68°C for 1 min. PCR products were purified using QIAquick PCR Purification Kit and analysed by restriction digestion and DNA sequencing.

### 2.8 RNA-seq

The total RNA was extracted with RNeasy Plus Mini Kit (Qiagen; 74134). MEF feeder cells were removed from ESC culture before RNA extraction by plating cells on a gelatine-coated dish for 30 min during the passaging and expanding unattached cells in a new dish. Library preparation was performed using the TruSeq stranded mRNA sample prep kit (Illumina; RS-122-2101) according to the manufacturer’s instructions. Sequencing was performed on the Illumina NovaSeq 6000 platform in a 100 bp paired-end mode. Sequenced reads were mapped to the mouse reference genome sequences (GRCm38/mm10) using TopHat v2.0.13^19^ in combination with Bowtie2 ver. 2.2.3^20^ and SAMtools ver. 0.1.19.^21^ The fragments per kilobase of exon per million mapped fragments (FPKMs) were calculated using Cufflinks version 2.2.1^22^ (Supplementary Table S1).

### 2.9 Data availability

The RNA-seq data are available in the DNA Data Bank of Japan Sequencing Read Archive under the accession number DRA013360.

## 3 Results

### 3.1 Overview of the methods for deleting the 2.5-Mb genomic region

**Figure 1.**
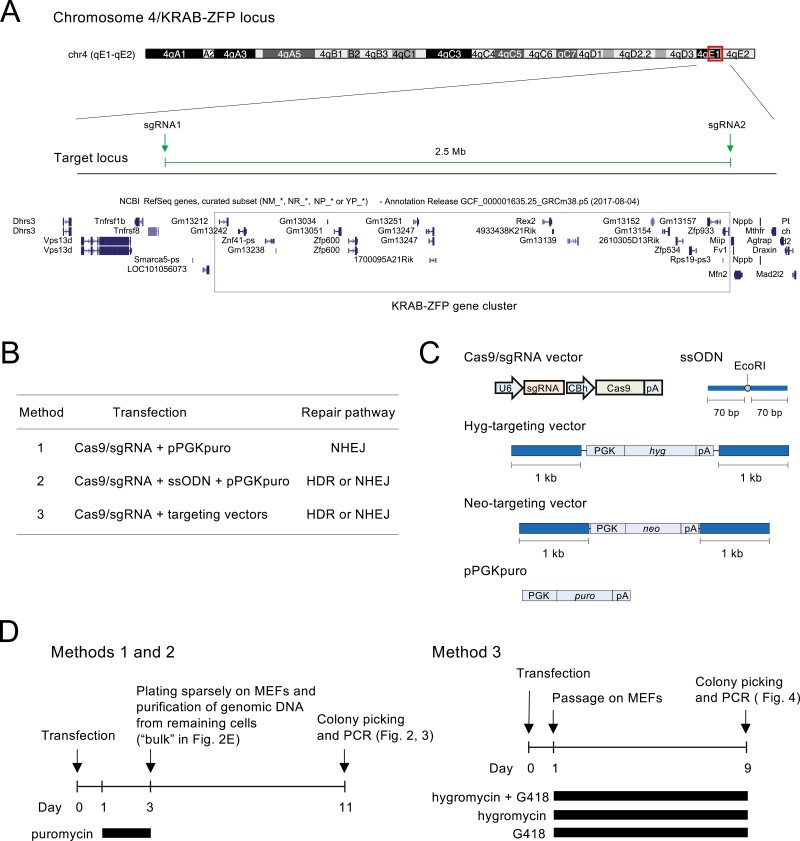
Protocols for inducing genomic deletion. (a) UCSC genome browser view of the KRAB-ZFP gene cluster and the position of the sgRNAs used for genomic deletion. (b) Summary of the three methods compared in this study. (c) Schematic of the vector structures. U6, U6 promoter; CBh, truncated CBA hybrid promoter; PGK, phosphoglycerate kinase-1 promoter; pA, polyadenylation signal. (d) Time course of Methods 1, 2, and 3.

Figure 1a shows the 2.5-Mb genomic region of the KRAB-ZFP gene cluster located on the distal side of mouse chromosome 4. We attempted to delete this entire region in mouse ESCs by cleaving the upstream and downstream sites with sgRNAs.

We compared three methods (Fig. 1b). In all methods, the plasmid vector pX330^18^ was used to express Cas9 and sgRNAs, and the TransFast transfection reagent, which employs lipid-mediated gene transfer, was utilized to introduce DNA into ESCs. In Method 1, repair template DNA was not transfected. Therefore, cleaved sites were repaired by NHEJ. In Method 2, a 146-base ssODN containing 70-base 5’and 3’ homology arms (Fig. 1c) was co-transfected as a repair template for HDR. We introduced an EcoRI site between the homology arms (Fig. 1c) to facilitate the identification of HDR events. It was expected that NHEJ would still be observed in Method 2 in case the ssODN was not utilized during repair. To enrich transfected ESCs, we co-transfected a puromycin resistance gene expression vector in Methods 1 and 2 (Fig. 1b) and selected ESCs using puromycin between 24 and 72 h after transfection (Fig. 1d, left). The ESCs were then sparsely plated on mitomycin C-treated MEF feeder cells. After 8 days of culture, ESC colonies were isolated and genomic deletions were screened by PCR (Fig. 1d, left). In Method 3, we transfected two double-stranded targeting vectors together with the Cas9/sgRNA expression vector (Fig. 1b and c). Each targeting vector contained the hyg gene and the neo gene, respectively. Both vectors contained the same 1-kb homology arms corresponding to the upstream and downstream regions of the genomic cleavage sites. We expected that co-transfection of two targeting vectors and selection for hygromycin/G418 double resistance would increase the efficiency of identifying biallelic deletions. One day after transfection, ESCs were split and selected with both hygromycin and G418, hygromycin only, and G418 only (Fig. 1d, right). Nine days after selection, drug-resistant colonies were isolated and genomic deletions were screened by PCR.

### 3.2 Comparison of genomic deletions with and without the ssODN

**Figure 2.**
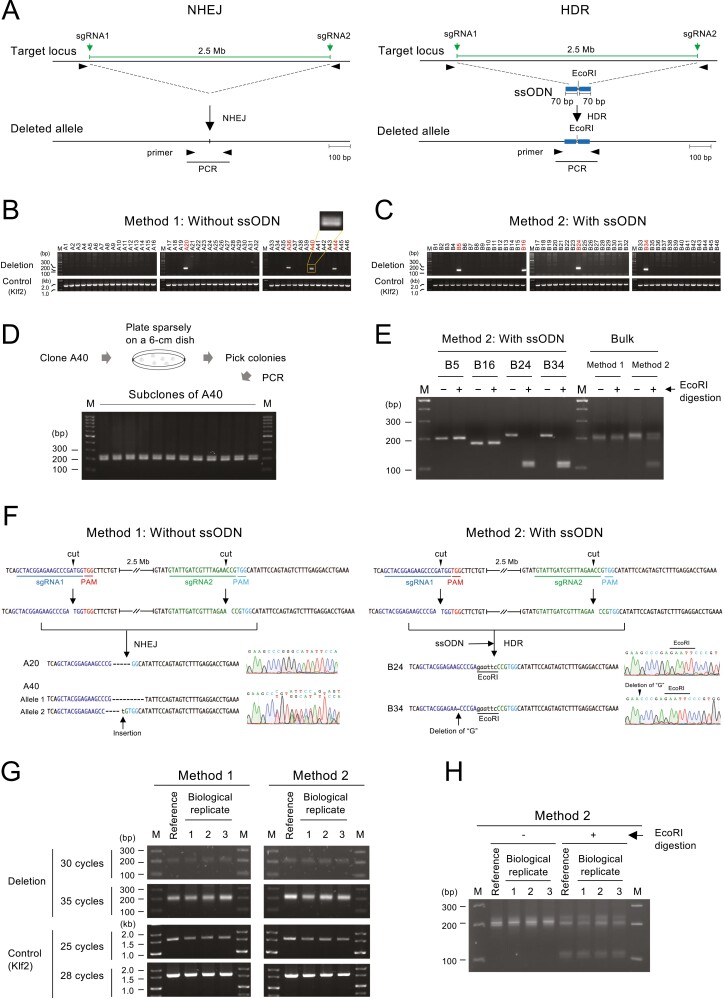
Genomic deletion induced by Method 1 and Method 2. (a) Predicted scheme of genomic deletions induced by NHEJ (left) and ssODN-mediated HDR (right). (b, c) PCR screening of genomic deletions in Method 1 (b) and Method 2 (c). A magnified view of clone A40 is shown to depict the two bands that are close in size. The Klf2 locus was used as an internal control for integrity and quantity of the template DNA. M, size marker. (d) Schematic diagram showing the procedure for subcloning A40 and the result of the PCR analysis. (e) EcoRI digestion of PCR products obtained in Method 2. (f) Representative results of the sequence analysis of the PCR products obtained in Method 1 (left) and Method 2 (right). Dashed lines indicate nucleotide deletions from the Cas9/sgRNA-mediated cleavage site. (g) Reproducibility of deletion efficiency by the analysis of the bulk cell population. Reference indicates the bulk cell populations shown in (e). (h) Reproducibility of the ratio of NHEJ and HDR by the analysis of the bulk cell population.

We compared Method 1, in which no repair template was introduced, and Method 2, in which an ssODN was introduced as a repair template. Single-cell–derived colonies were isolated according to the protocol in Fig. 1d, and genomic deletions were detected by PCR as shown in Fig. 2a. Both HDR and NHEJ were expected to occur in Method 2. We distinguished them by digesting the PCR products with EcoRI (Fig. 2a, right).

We observed bands of the expected size in an agarose gel after PCR (Fig. 2b and c). In both Method 1 and Method 2, 4 out of 46 colonies (9%) were PCR-positive (Fig. 2b and c). In Method 1, two bands of similar size were observed in one lane (A40 in Fig. 2b). To exclude the possibility that two clones were fused during ESC colony formation, PCR was conducted after recloning (Fig. 2d). However, the two bands were observed even after recloning. Therefore, we concluded that these two bands derive from a single clone. Their presence suggests the possibility that deletions occurred in both alleles. We address this point later in Fig. 5.

To compare the efficiency of HDR and NHEJ in Method 2, the four PCR products obtained in Fig. 2c were digested by EcoRI. EcoRI cleavage was observed in two of the products (Fig. 2e), indicating that the efficiency of HDR and NHEJ was comparable. To further assess this observation, we purified genomic DNA from the bulk cell population (Fig. 1d), conducted PCR to amplify deletion junctions, and digested the PCR products with EcoRI (Fig. 2e). As expected, the PCR product obtained by Method 1 was not cleaved by EcoRI. On the other hand, EcoRI-cleaved bands were observed in the products derived by Method 2, and the intensity of cleaved and uncleaved bands was similar. Thus, as in the analysis of cloned cells, the efficiency of HDR and NHEJ was considered comparable.

To confirm that the PCR amplification represented the deletion of the targeted gene cluster, we sequenced the PCR products (Fig. 2f). Two clones derived by both Method 1 and Method 2 were analysed, and the results confirmed that all PCR products represented the deletion of the targeted gene cluster. Both PCR products from Method 2 were cleaved with EcoRI (Fig. 2e), suggesting that the deletion was completed in a precise manner. Sequence analysis revealed that one of the PCR products had a single base deletion of a guanine nucleotide upstream of the EcoRI site (B34 in Fig. 2f) (see Section 4).

Although we enriched transfected cells by transient puromycin selection (Fig. 1d), PCR bands were not detected in more than 90% of the clones (Fig. 2b and c). This observation suggests the possibility that the transfected cells were inefficiently enriched, resulting in a low deletion rate. Therefore, we PCR-amplified the upstream and downstream sgRNA target sites for three clones in both Methods 1 and 2 and examined whether sgRNA target sites were mutated by direct sequencing (Supplementary Fig. S1). We detected indels in both sgRNA target sites in two clones in Method 1 and one clone in Method 2, as well as in one of the sgRNA target sites in one clone in both Methods. No mutation was detected in either sgRNA target sites in one clone in Method 2. The absence of a mutation at the sgRNA target site does not mean that the cells were not transfected because the cleaved end may be repaired correctly. Therefore, the result indicates that at least around half of the cells that survived transient puromycin selection were transfected with the Cas9/sgRNA expression vectors.

To verify our observation that the efficiency of HDR and NHEJ was comparable, we further conducted three independent transfections for Methods 1 and 2, purified genomic DNA from the bulk cell population, and performed semi-quantitative PCR by changing the number of amplification cycles (Fig. 2g). The intensity of the PCR band was similar between new transfections (‘Biological replicates 1–3’ in Fig. 2g) and the transfection analysed in Fig. 2b–f (‘Reference’ in Fig. 2g). Furthermore, the intensity of the EcoRI-cleaved and -uncleaved band was also similar in all transfections (Fig. 2h). These results demonstrate the reproducibility of the deletion experiment and reinforce our conclusion that the efficiencies of HDR and NHEJ are similar.

### 3.3 Genomic inversions by NHEJ

**Figure 3.**
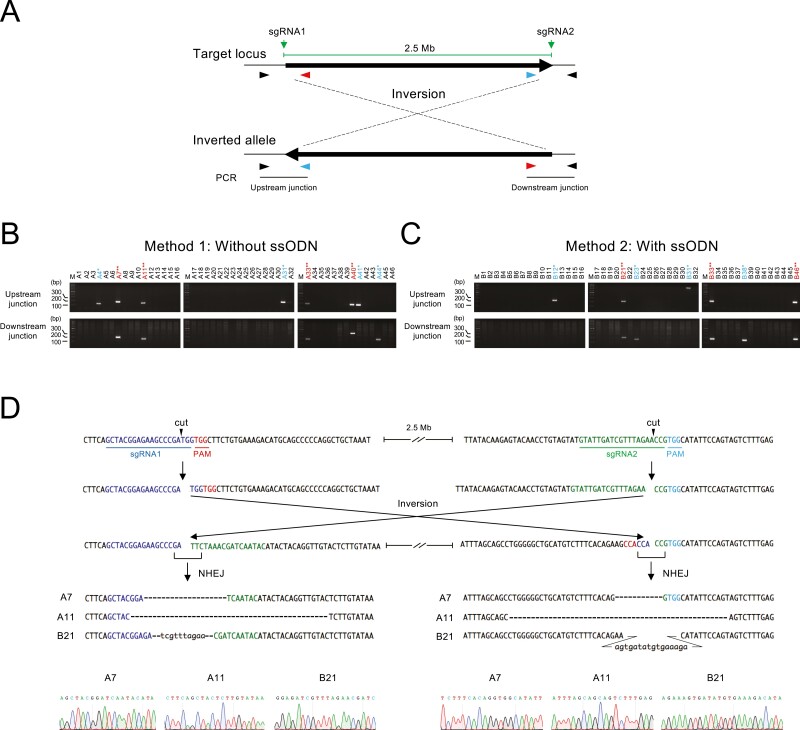
Genomic inversion induced by NHEJ. (a) Predicted scheme of genomic inversion induced by NHEJ. (b, c) PCR screening of genomic inversions in Method 1 (b) and Method 2 (c). Clones that are PCR-positive in either upstream or downstream junction are indicated by an asterisk, and clones PCR-positive in both junctions are marked by double asterisks. (d) Sequence analysis of the PCR products obtained in (b) and (c).

Megabase-scale genomic inversion has been reported in mice after microinjection of Cas9 and gRNAs into zygotes.^8^ Side-by-side comparison of megabase-scale inversion and deletion frequencies in cultured cells would be useful for chromosomal engineering and generation of human disease models. Therefore, we investigated whether inversion of the 2.5-Mb KRAB-ZFP gene cluster occurred in Methods 1 and 2 (Fig. 3). PCR primers were set at each sgRNA target site so that the junction of inversions was amplified (Fig. 3a). In Method 1, 7 and 5 out of 46 clones were PCR-positive at the upstream and downstream junctions, respectively (Fig. 3b). Among them, four clones were PCR-positive at both the upstream and downstream junctions (Fig. 3b). In Method 2, 5 and 5 out of 46 clones were PCR-positive at the upstream and downstream junctions, respectively, and 3 clones were PCR-positive at both junctions (Fig. 3c). We sequenced the PCR fragment in three clones, two from Method 1 and one from Method 2, which were PCR-positive in both junctions, and confirmed that the inversion had occurred (Fig. 3d). These results indicate that the frequency of megabase-scale inversion was comparable to megabase-scale deletion.

### 3.4 Genomic deletions using targeting vectors

**Figure 4.**
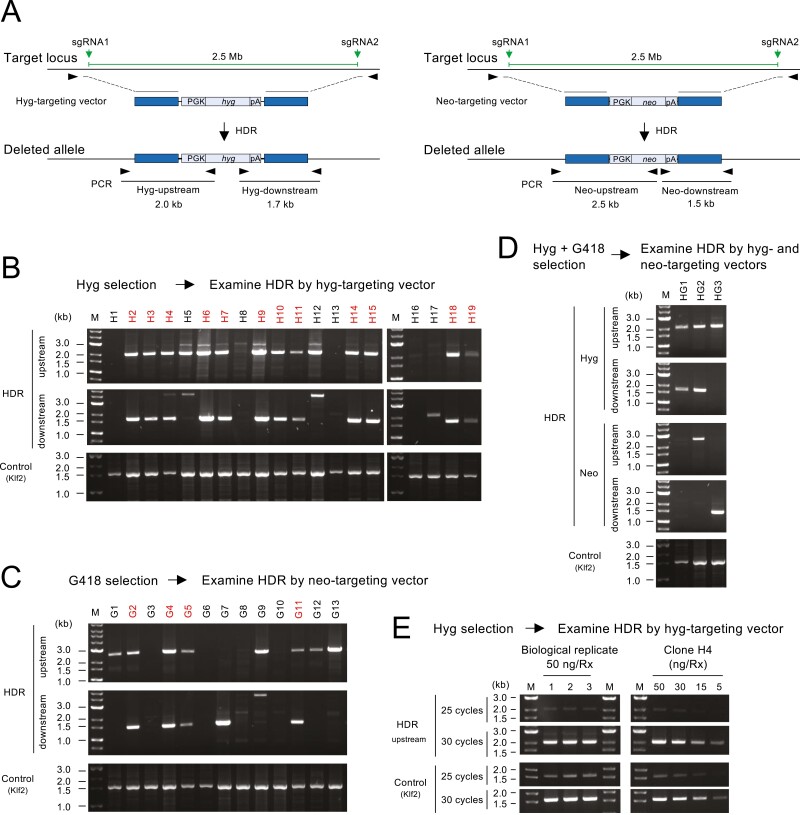
Genomic deletion induced by Method 3. (a) Predicted scheme of genomic deletions induced by HDR following transfection of targeting vectors. Two targeting vectors, each containing the hyg and the neo cassette, were co-transfected. (b–d) PCR screening of genomic deletions in hygromycin-resistant clones (b), G418-resistant clones (c), and hygromycin/G418 double-resistant clones (d). The Klf2 locus was PCR-amplified as an internal control to examine the integrity and the quantity of the template DNA. M, size marker. (e) Reproducibility of deletion efficiency by the analysis of the bulk cell population. Rx, reaction.

Next, we attempted genomic deletion by Method 3, which involves the transfection of hyg- and neo-targeting vectors for HDR. Following transfection, ESCs were selected with both hygromycin and G418, hygromycin only, or G418 only (Fig. 1d). Drug-resistant clones were analysed by PCR using two primer pairs that detect HDR in the upstream and downstream regions (Fig. 4a).

First, we analysed hygromycin-resistant clones. Out of the 19 clones analysed, the expected recombination was observed in both upstream and downstream regions in 12 clones (63%; Fig. 4b). Next, we analysed 13 G418-resistant clones and observed the expected recombination in both upstream and downstream regions in 4 clones (31%; Fig. 4c). Thus, the mean deletion efficiency of HDR using targeting vectors was 47%, which is five times higher than that of NHEJ or ssODN-mediated HDR. Finally, we analysed hygromycin/G418 double-resistant clones to investigate whether they harbour a biallelic mutation. We obtained much fewer colonies via hygromycin/G418 double selection compared to the single selections. We analysed three double-resistant clones by PCR using four primer sets for each clone; however, the expected recombination was not observed with at least one of the primer sets (Fig. 4d), suggesting that biallelic deletion is not a frequent event.

To confirm the reproducibility of the deletion efficiency by HDR, we conducted three independent transfections for Method 3, purified genomic DNA from the bulk cell population, and performed semi-quantitative PCR using primers detecting the upstream recombination junction (Fig. 4e). Clone H4, one of the targeted clones shown in Fig. 4b, was also included for reference. The efficiency of PCR was similar between three transfections (‘Biological replicates 1–3’ in Fig. 4e) and clone H4, demonstrating the reproducibility of the deletion experiment.

### 3.5 Comparison of biallelic deletion frequency between the three methods

To analyse the phenotypes caused by genomic deletions, it is often necessary to introduce deletions in both alleles. However, the results of the analysis of hygromycin/G418 double-resistant clones suggested that biallelic deletion is not frequent (Fig. 4d). Therefore, we systematically compared the frequency of biallelic deletion among the three methods by analysing the PCR-positive clones shown in Figs. 2 and 4.

**Figure 5.**
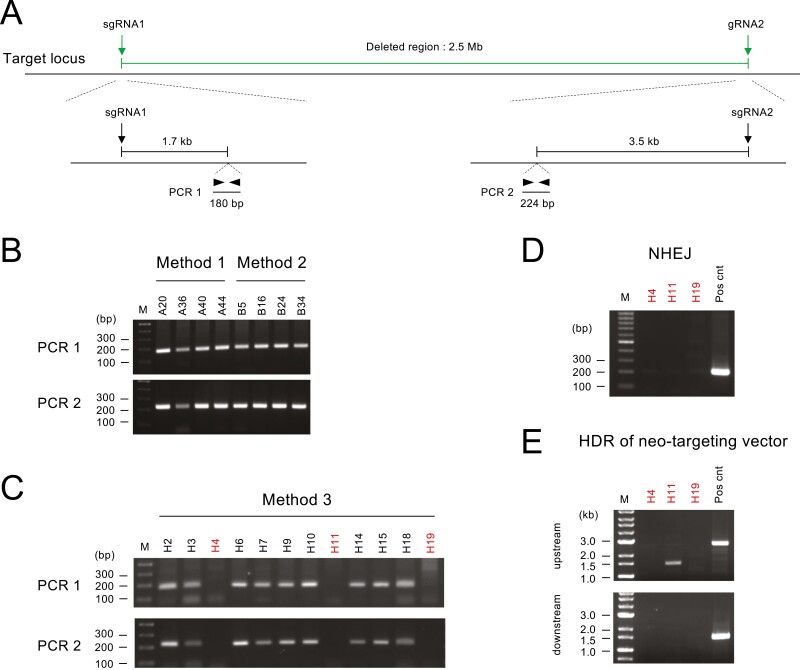
Identification of biallelic deletion events. (a) Location of the PCR primers for the screening of biallelic deletion. (b, c) PCR screening for biallelic deletion of the candidate clones obtained by Method 1 and Method 2 (b) and Method 3 (c). (d) Screening for NHEJ-mediated genomic deletion. The same PCR protocol as in Fig. 2b was performed. Clone A20, which was PCR-positive in Fig. 2b, was used as a positive control as indicated (Pos cnt). (e) Screening for HDR mediated by the neo-targeting vector. The same PCR protocol as in Fig. 4c was performed. Clone G2, which was PCR-positive in Fig. 4c, was used as a positive control (Pos cnt).

We set up two pairs of PCR primers within the deleted region (Fig. 5a). If both alleles were deleted, no amplification should be detected. For Methods 1 and 2, we analysed all clones that showed deletion in Fig. 2. PCR amplification was observed in all clones (Fig. 5b), suggesting that biallelic deletion did not occur. This includes the clone A40, which showed two bands in Fig. 2b and d (see Section 4). For Method 3, we analysed 12 hygromycin-resistant clones in which the predicted recombination was observed in both upstream and downstream regions (Fig. 4b). No amplification was observed in three clones (H4, H11, H19; Fig. 5c), suggesting that both alleles were deleted in these clones. To investigate whether biallelic deletion accompanied NHEJ, which does not involve the recombination of the targeting vector as observed in Method 1, we conducted the same PCR analysis performed in Fig. 2b. No amplification was detected (Fig. 5d), suggesting that either the NHEJ observed in Method 1 did not occur or that NHEJ with the deletion of the binding site of the PCR primer occurred. To examine the possibility that biallelic deletion involved HDR by the neo-targeting vector, we conducted the same PCR analysis shown in Fig. 4c. No amplification was detected in two of the three biallelic mutants (Fig. 5e). Although PCR amplification was detected in one of the mutants in the analysis of the upstream region (clone H11), the band size was different from the expected one and no amplification was detected in the downstream region (Fig. 5e), suggesting that HDR by the neo-targeting vector did not occur. On the basis of the results of Fig. 5d and e, we speculate that biallelic deletion was introduced through biallelic HDR by the hyg-targeting vector or through the combination of single-allele HDR by the hyg-targeting vector and NHEJ accompanied by the deletion of the PCR primer binding site.

### 3.6 SNP analysis of the allele heterozygosity outside the expected deletion region

**Figure 6.**
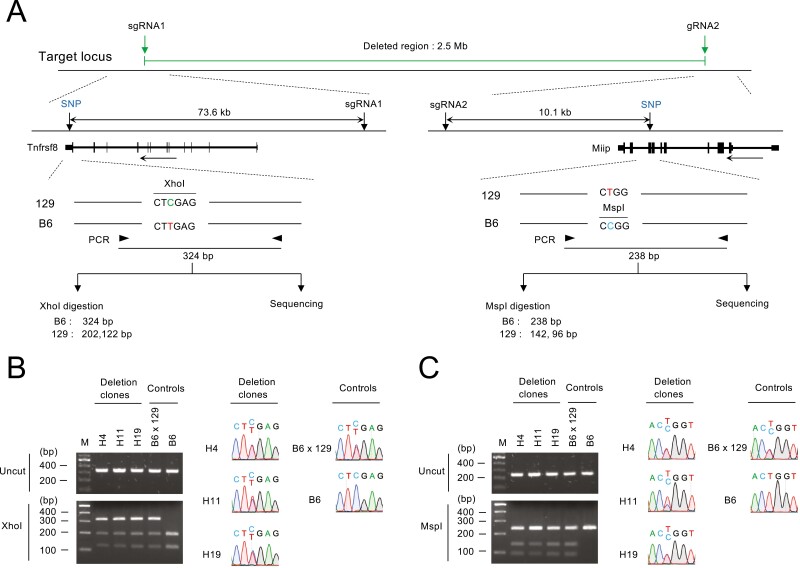
SNP analysis of deletion mutants. (a) Schematic diagram of the SNP analysis upstream and downstream of the deletion region. Horizontal arrows below the structure of the Tnfrsf8 and Miip genes indicate direction of the transcription. Note that the parental ESC line of the deleted clones is the F1 hybrid of C57BL/6 and 129/Sv mouse strains. B6, C57BL/6; 129, 129/Sv. (b, c) SNP analysis of biallelic deletion clones by restriction digestion and DNA sequencing.

Recent reports have shown that CRISPR-Cas9-mediated double-strand breaks (DSBs) can give rise to massive structural changes in chromosomes, such as large mono-allelic deletions and loss of heterozygosity of entire chromosome arms.^23–26^ However, PCR analysis of the junction at the vector insertion site (Figs. 4 and 5) does not detect such massive chromosomal changes. To investigate whether unintended massive chromosomal changes occurred, we analysed biallelic deletion clones for the heterozygosity at the upstream and downstream of the expected deletion site using SNPs detection (Fig. 6a). The parental ESC line of the deleted clones is an F1 hybrid derived from C57BL/6 and 129/Sv mouse strains,^14^ and thus SNPs are present throughout the chromosome. Tnfrsf8 and Miip are the closest genes to the upstream and downstream sgRNA cleavage sites, respectively (Fig. 6a). We identified an SNP containing a restriction digestion site within each gene. We then amplified the genomic region containing the SNP by PCR and checked for heterozygosity by restriction digestion and Sanger sequencing. We detected heterozygous SNPs both upstream and downstream of the target cut site in all clones (Fig. 6b and c), indicating that massive structural changes in the chromosomes did not occur.

### 3.7 RNA-seq analysis of deletion mutants

**Figure 7.**
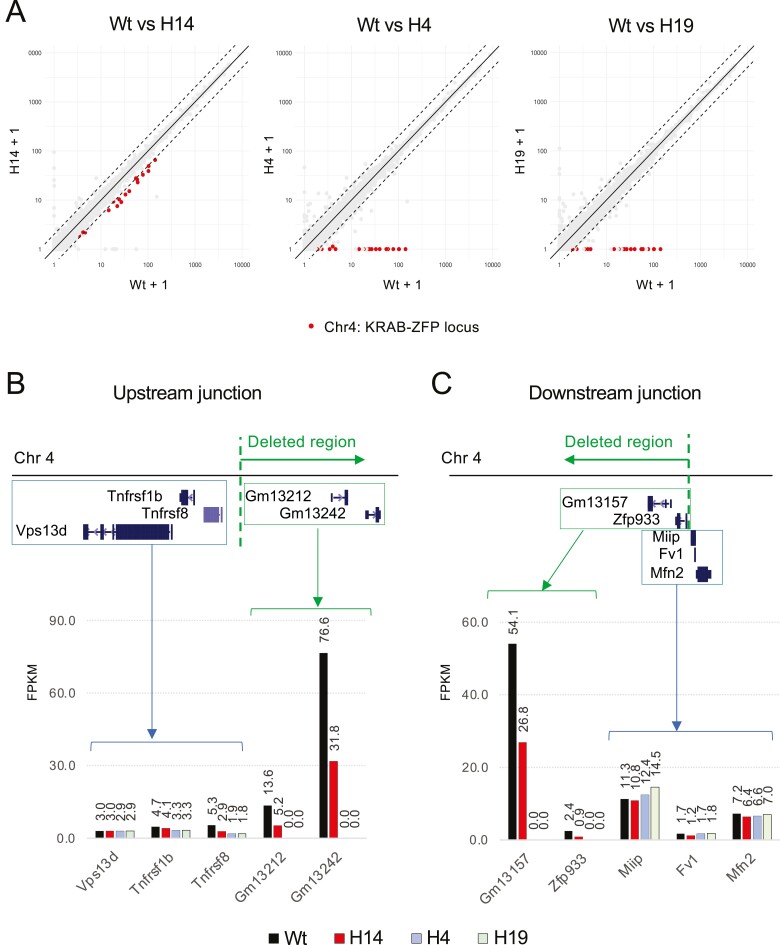
RNA-seq analysis of deletion mutants. (a) Expression analysis of the KRAB-ZFP gene cluster. The gene expressions of the single-allele deletion mutant (H14) and biallelic deletion mutants (H4 and H19) were compared with wild-type ESCs (Wt). Data are shown in FPKM. (b, c) Gene expression at the upstream (b) and downstream (c) deletion junctions. Note that the gene expression outside the deletion target site was detectable in both biallelic deletion mutants (H4 and H19), indicating that biallelic deletion was confined to the predicted region.

To further validate the deletion of the 2.5-Mb region, we performed RNA-seq in the following four cell lines—wild-type ESCs, the clone H14 with single-allele deletion, and the clones H4 and H19 with biallelic deletion—and compared the gene expression at the deletion target site (Fig. 7, Supplementary Table S1). In the single-allele deletion clone (H14), the expression of the gene cluster within the deletion target site was reduced by approximately 2-fold compared with wild-type ESCs, as predicted (Fig. 7a, left). By contrast, in the biallelic deletion clones (H4 and H19), the expression of the gene cluster was almost undetectable (Fig. 7a, middle and right), confirming the biallelic deletion of the 2.5-Mb gene cluster. We then analysed the gene expression around the upstream (Fig. 7b) and the downstream (Fig. 7c) deletion junctions. The gene expression was detectable outside the deletion target site in both biallelic mutants (Fig. 7b and c), indicating that the deletion was confined to the expected region. These results demonstrate that the biallelic deletion of the 2.5-Mb gene cluster was achieved using the targeting vectors.

## 4. Discussion

In this study, we compared three megabase-scale genomic deletion methods in mouse ESCs: Method 1 using Cas9/sgRNA only, Method 2 using Cas9/sgRNA and ssODN, and Method 3 using Cas9/sgRNA and targeting vectors. The results showed that all methods are feasible at least for mono-allelic deletion. On the other hand, the three methods differed in the simplicity of the experimental design and the efficiency of deletion. Therefore, the choice of method depends on the purpose of the experiment. In the following section, we compare the three methods and discuss some considerations to further improve deletion efficiency.

The deletion efficiency in Methods 1, 2, and 3 were 9%, 9%, and 31–63%, respectively. Furthermore, biallelic deletion was observed only in Method 3. This indicates that Method 3, which uses a targeting vector, is superior to the others when considering only the efficiency of deletion. However, there are some drawbacks to using targeting vectors. First, generating a targeting vector is time consuming. Second, setting up experimental conditions for PCR screening of the deletion clones may take time compared to Methods 1 and 2 because a longer PCR amplification is required. Therefore, Methods 1 and 2, which are straightforward in their experimental design, may be sufficiently effective if the number of clones to be screened by PCR is manageable. In fact, a recent report demonstrated biallelic deletion of the same KRAB-ZFP gene cluster by a procedure similar to Method 2.^12^ Although the efficiency of biallelic deletion is not described in this report, the results suggest that sufficient deletion efficiency may be achieved without using a targeting vector by optimizing experimental conditions. Notably, we detected inversion of the 2.5-Mb region at similar efficiency to the deletion (Fig. 3). Chromosomal inversions are observed in human disease.^27^ Our result indicates that human disease modelling by chromosomal inversion is feasible in cultured cells.

Several possible improvements can be made to increase the efficiency of megabase-scale genomic deletion. The first is to use a ribonucleoprotein (RNP) complex consisting of Cas9 and sgRNA. A recent report demonstrated that transfection of a Cas9/sgRNA RNP complex was more efficient in cleaving the genome than transfection of Cas9/sgRNA expression vectors. The authors argue that the intracellular assembly of Cas9 and sgRNAs expressed from transfected vectors is hampered by the competitive binding of mRNA to Cas9.^28^ Second, the optimization of transfection conditions may significantly affect the deletion efficiency. In the same report described above, two electroporators, MaxCyte and 4D-Nucleofector, were compared for the introduction of mutations into human iPSCs by ssODNs.^28^ The results showed that MaxCyte was superior to 4D-Nucleofector in terms of mutagenesis efficiency. In our study, cationic lipid-based transfection reagents were used. The use of other transfection methods may improve the efficiency of megabase-scale genomic deletions. Third, the use of single-stranded targeting vectors may be useful. Previous studies in zygote microinjection have suggested that long single-stranded DNA donors are efficient templates for HDR.^29–31^ However, targeting vectors used in cultured cells are usually several kb in length because of the presence of a selection marker cassette, and the preparation of such a long single-stranded DNA of high quality is labour intensive. Recently, it has become possible to synthesize long single-stranded DNA commercially, which may apply to megabase-scale deletion. Fourth, improved Cas9 with higher cutting efficiency^32^ and gRNA design with higher editing efficiency^33^ would be useful to increase the overall efficiency of genome editing. Fifth, optimizing the amount of gRNA expression vectors and the donor DNAs may increase the efficiency of megabase-scale deletion.

It has been reported that a DSB in CRISPR-Cas9 genome editing induces unexpectedly large structural changes in chromosomes, such as large deletions and chromosome loss.^23–26^ Such unexpected chromosomal changes may occur more frequently in the present study because two DSBs need to be introduced to achieve the megabase-scale deletion. Therefore, we analysed allelic heterozygosity around the deletion region in three biallelic deletion clones using flanking SNPs. We found that allelic heterozygosity was maintained in all clones, suggesting that no large-scale changes in chromosome structure occurred. However, the frequency of such unexpected chromosomal changes may vary from cell to cell and locus to locus. In fact, studies using human iPSCs have shown that large chromosomal changes such as mono-allelic genomic deletions and loss of heterozygosity were observed in up to 40% of CRISPR-edited clones.^25^ Such chromosomal changes may be missed by standard screening procedures in genome editing such as PCR amplification and sequencing of the edited locus. Such structural variation can be reliably detected using quantitative genomic PCR and SNP genotyping, both of which are simple and low-cost assays.^25^ The F1 hybrid ESCs in the current study^14^ are rich in SNPs that distinguish sequence differences between alleles and are useful in analysing heterozygosity.

A recent report demonstrated that DSBs introduced in CRISPR-Cas9 genome editing can give rise to chromothripsis-massive, clustered genomic rearrangements observed in cancer and human congenital diseases.^34^ The possibility of causing chromothripsis demands added caution when interpreting the results of genome editing. Indeed, the PCR analysis of the clone A40 cannot be simply explained (Figs. 2b and d and 5b). This clone showed two bands during screening for genomic deletions (Fig. 2b) and even after recloning (Fig. 2d), suggesting that biallelic deletion may have occurred. However, further analysis revealed that the target site was retained in the genome (Fig. 5b). One possible interpretation of this observation is that the DSBs may have induced a complex genomic rearrangement and duplication of the target site sequence. Since the extent of the genomic rearrangement induced by chromothripsis cannot be predicted, genome-wide quality control of the edited clones, such as whole genome sequencing or RNA-seq as conducted in Fig. 7, may be called for depending on the purpose of the experiment.

PCR analysis of the positive clone obtained by Methods 2 revealed a single base pair deletion upstream of the EcoRI site (B34 in Fig. 2f). This is not the result of a PCR error because this sequence was obtained by direct sequencing of the PCR product. Since oligonucleotide synthesis is not perfectly accurate, this deletion may have been pre-existing in the ssODN. It is also possible that the ssODN sequence was accurate but the HDR in the megabase-scale deletion was incomplete.

Although we could achieve biallelic deletion of the 2.5-Mb target region, we could not determine whether the biallelic deletion was introduced through biallelic HDR by the hyg-targeting vector or through the combination of single-allele HDR and NHEJ accompanied by the deletion of the PCR primer binding site. To distinguish these possibilities, digital PCR would be useful. Digital PCR allows absolute quantification of the target DNA without the standard curve of reference DNA.^35^ If two copies of the hyg-targeting vector per genome are detected by digital PCR, the possibility of random insertion of the vector is not excluded. However, if one copy of vector DNA per genome is detected by digital PCR, we can conclude that the biallelic mutation was caused by the combination of HDR and NHEJ.

It should be noted that the expression of Tnfrsf8 was reduced in the single allele and the biallelic deletion clones compared to wild-type ESCs (Fig. 7b). This may suggest that the regulatory element of the Tnfrsf8 gene is located in the deleted region. Another possibility is that the insertion of the hyg cassette into the deletion site affects the expression of the Tnfrsf8 gene. This is because bacterial sequences have been reported to cause the silencing of adjacent genes when introduced into animal cells.^36^ This possibility can be addressed by examining the expression of the Tnfrsf8 gene in clones obtained by Methods 1 or 2.

Taken together, the results of this study will serve as a benchmark for selecting methods to introduce megabase-scale genomic deletions.

## Supplementary Material

dsac045_suppl_Supplementary_Figure_S1

dsac045_suppl_Supplementary_Table_S1

dsac045_suppl_Supplementary_Table_S2
