# Supplementary material for: Comparison of CRISPR-Cas9-mediated megabase-scale genome deletion methods in mouse embryonic stem cells
Source: DNA Res. 2022 Nov 30;30(1):dsac045. doi: 10.1093/dnares/dsac045 (PMC9847339; doi:10.1093/dnares/dsac045)
Supplement: dsac045_suppl_Supplementary_Figure_S1 [file dsac045_suppl_supplementary_figure_s1.pdf]

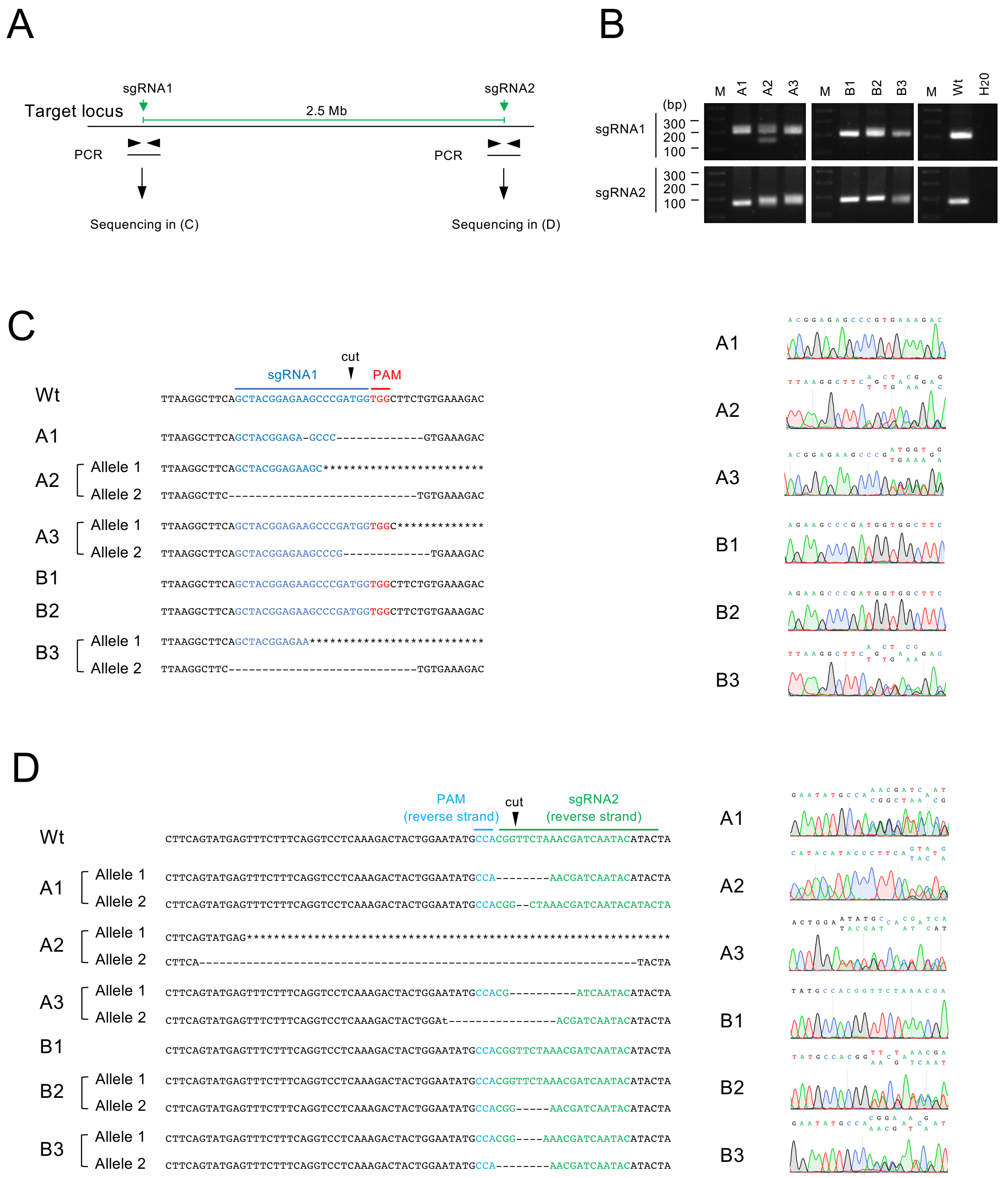

Supplementary Figure 1. Indels of sgRNA target sites.

(A) PCR primer positions used for screening indels. (B) PCR products at the sgRNA target sites. Clone names correspond to those in Figure 2. M, DNA size marker. (C, D) Sequence analysis of the PCR products at the upstream (C) and downstream (D) sgRNA target sites. Dashed lines indicate nucleotide deletions. The sequence of the regions marked with asterisks could not be determined due to sequence overlap from different alleles. The lowercase letter in allele 2 of clone A3 indicates nucleotide insertion.
